# Evidence-Based Malaria Control and Elimination in the Amazon: Input from the International Center of Excellence in Malaria Research Network in Peru and Brazil

**DOI:** 10.4269/ajtmh.21-1272

**Published:** 2022-10-13

**Authors:** Marcelo U. Ferreira, Dionicia Gamboa, Katherine Torres, Hugo Rodriguez-Ferrucci, Veronica E. Soto-Calle, Karim Pardo, Pablo S. Fontoura, Sheena S. Tomko, Ricardo T. Gazzinelli, Jan E. Conn, Marcia C. Castro, Alejandro Llanos-Cuentas, Joseph M. Vinetz

**Affiliations:** ^1^ Department of Parasitology, Institute of Biomedical Sciences, University of São Paulo, São Paulo, Brazil;; ^2^ Institute of Tropical Medicine Alexander von Humboldt, Universidad Peruana Cayetano Heredia, Lima, Peru;; ^3^ Laboratorios de Investigación y Desarrollo, Facultad de Ciencias y Filosofía, Universidad Peruana Cayetano Heredia, Lima, Peru;; ^4^ Facultad de Medicina Humana, Universidad Nacional de la Amazonia Peruana, Iquitos, Peru;; ^5^ Dirección de Prevención y Control de Enfermedades Metaxénicas y Zoonosis, Ministerio de Salud, Lima, Peru;; ^6^ Universidad de Ciencias Aplicadas and Ejecutiva Adjunta II, Despacho Viceministerial de Salud Pública, Ministerio de Salud, Lima, Peru;; ^7^ Coordenação-Geral de Arboviroses, Secretaria de Vigilância em Saúde, Ministério da Saúde, Brasília, Brazil;; ^8^ Department of Biology, University of Pennsylvania, Philadelphia, Pennsylvania;; ^9^ Instituto de Pesquisas Rene Rachou, Fundação Oswaldo Cruz, Belo Horizonte, Brazil;; ^10^ Division of Infectious Disease and immunology, Department of Medicine, University of Massachusetts Medical School, Worcester, Massachusetts;; ^11^ Plataforma de Medicina Translacional, Fundação Oswaldo Cruz, Ribeirão Preto, Brazil;; ^12^ Department of Biomedical Sciences, School of Public Health, University at Albany, State University of New York, Albany, New York;; ^13^ Wadsworth Center, New York State Department of Health, Albany, New York;; ^14^ Department of Global Health and Population, Harvard T. H. Chan School of Public Health, Boston, Massachusetts;; ^15^ Section of Infectious Diseases, Department of Internal Medicine, Yale School of Medicine, New Haven, Connecticut

## Abstract

Malaria remains endemic in 17 countries in the Americas, where 723,000 cases were reported in 2019. The majority (> 90%) of the regional malaria burden is found within the Amazon Basin, which includes nine countries and territories in South America. Locally generated evidence is critical to provide information to public health decision makers upon which the design of efficient and regionally directed malaria control and elimination programs can be built. *Plasmodium vivax* is the predominant malaria parasite in the Amazon Basin. This parasite species appears to be more resilient to malaria control strategies worldwide. Asymptomatic *Plasmodium* infections constitute a potentially infectious reservoir that is typically missed by routine microscopy-based surveillance and often remains untreated. The primary Amazonian malaria vector, *Nyssorhynchus* (formerly *Anopheles*) *darlingi*, has changed its behavior to feed and rest predominantly outdoors, reducing the efficiency of core vector control measures such as indoor residual spraying and distribution of long-lasting insecticide-treated bed nets. We review public health implications of recent field-based research carried out by the Amazonia International Center of Excellence in Malaria Research in Peru and Brazil. We discuss the relative role of traditional and novel tools and strategies for better malaria control and elimination across the Amazon, including improved diagnostic methods, new anti-relapse medicines, and biological larvicides, and emphasize the need to integrate research and public health policymaking.

## INTRODUCTION

The past decade has seen significant progress toward malaria elimination in the Americas. Of note, Paraguay, Argentina, and El Salvador were certified malaria-free by the WHO in 2018, 2019, and 2021, respectively. However, malaria remains endemic in 17 countries and territories in the region; 723,000 cases (76% of them resulting from *Plasmodium vivax*) were reported in 2019, and an estimated 139 million people remain at risk of locally acquired infection.^1^ The Amazon contributes 90% of the malaria burden in the Americas, with more intense transmission in riverine villages, farming settlements, gold mining camps, and Amerindian reserves.^2^ Together, Brazil and Peru account for 31% of all malaria cases in the Americas.^1^

There is no one-size-fits-all global strategy for malaria control and elimination. Human populations worldwide differ in levels of exposure and susceptibility to the five malaria parasite species transmitted to humans by 70 different species of *Anopheles* and *Nyssorhynchus* mosquitoes. Because sub-Saharan Africa and South and Southeast Asia account disproportionately for the worldwide disease burden of malaria, including the vast majority of malaria-attributable deaths,^1^ Amazonian malaria remains a relatively low-priority topic on the global public health agenda. Thus, locally generated evidence plays a critical role in the design of elimination strategies tailored for malaria-endemic settings across the Amazon.

The Amazonia International Center of Excellence in Malaria Research (ICEMR) program started in 2010 and currently involves investigators from five research institutions in the United States, one in Peru, and three in Brazil, in addition to a range of public health professionals from the Ministries of Health of Peru and Brazil. The program is funded by the National Institute of Allergy and Infectious Diseases, NIH. Key contributions from the Amazonia ICEMR network and collaborators in Peru and Brazil, with clear implications for the regional malaria elimination agenda, are highlighted in Table 1. These include improved case-finding strategies, novel field-deployable laboratory diagnosis, monitoring of antimalarial treatment efficacy and testing of new antimalarials, and tools for integrated vector control management.

**Table 1:** Challenges for malaria control in the Amazon and their public health implications

Challenge	Evidence from field studies in Amazonian Peru and Brazil	Public health implications
Case finding	1) Extensive asymptomatic parasite reservoir that remains undetected and untreated. 2) Asymptomatic *Plasmodium vivax* carriers are infectious to local vectors. 3) Reactive case detection–based strategies can identify a large proportion of parasite carriers that would be missed by passive case finding.	Active case-finding strategies are needed to eliminate the infectious human reservoir.
Laboratory diagnosis	1) Low-density parasitemias are common in low-endemicity settings and are typically missed by microscopy. 2) Rapid diagnostic test sensitivity for *Falciparum* malaria detection may be reduced drastically as a result of *hrp2* gene deletion. 3) Field-deployable molecular tests can detect submicroscopic parasitemias.	Infections are often missed by conventional microscopy and rapid diagnostic tests.
Treatment	1) First-line chloroquine–PQ treatment remains efficacious for *P. vivax* malaria, but > 10% of infections relapse despite routinely prescribed PQ treatment, possibly because of poor adherence and low cytochrome P450 2D6–mediated PQ metabolization. 2) Artemisinin-based combination therapies (artesunate–mefloquine and artemether–lumefantrine) remain highly efficacious for *P. falciparum* malaria. 3) Tafenoquine is as effective as low-dose PQ to prevent *P. vivax* relapses.	Malaria treatment regimens may be suboptimal despite their high efficacy when administered under supervision in clinical trials.
Vector control	1) Local vectors have shifted to predominantly exophagic and exophilic behavior. 2)* Nyssorhynchus darlingi* blood-feeding is mostly crepuscular. 3) Areas with accessible breeding habitats may benefit from larval source management strategies (e.g., larviciding).	Changes in biting behavior may undermine the efficacy of core vector control measures (indoor residual spraying and long-lasting insecticide-treated net distribution).

*hrp2* = histidine-rich protein 2; PQ = primaquine.

## POLICY IMPLICATIONS OF LOCALLY GENERATED EVIDENCE

**Figure 1.**
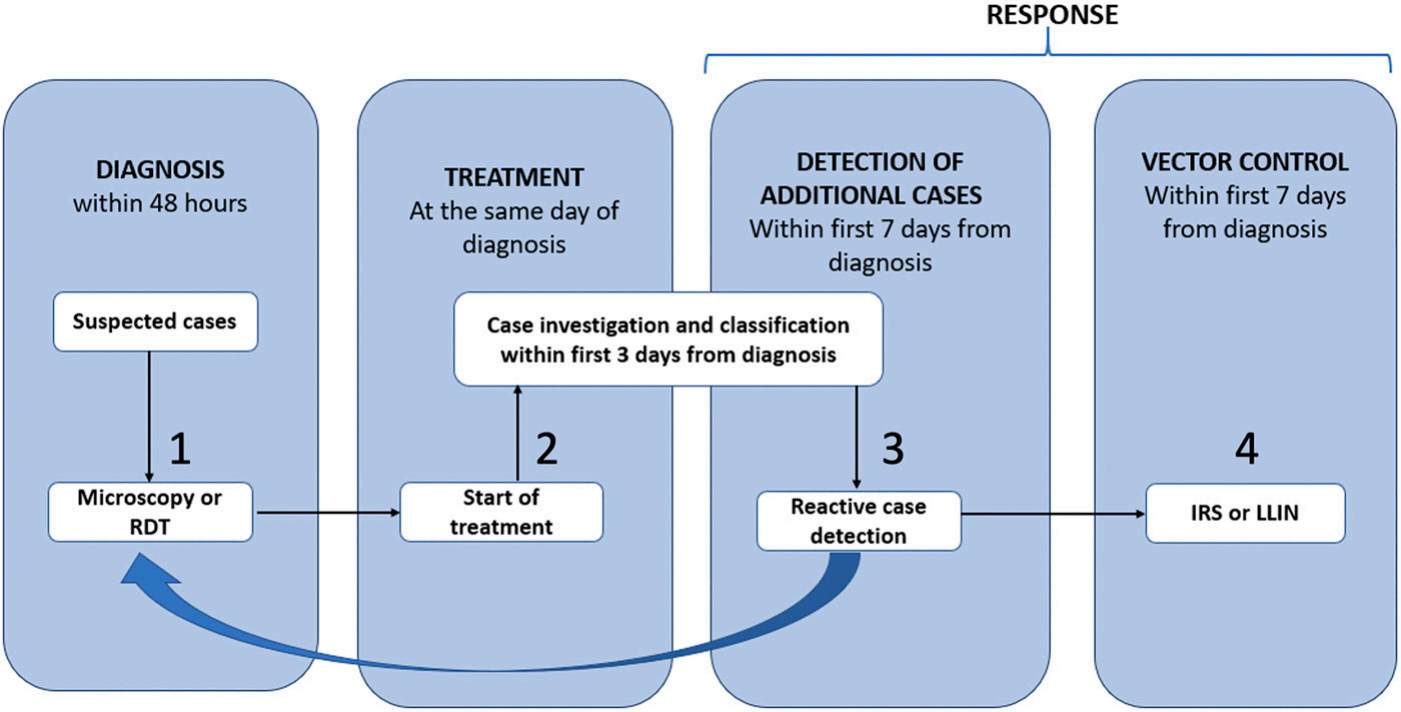
The Diagnosis, Treatment, Investigation, and Response strategy of the Pan American Health Organization for malaria control and elimination in the Americas. ILS = indoor residual spraying; LLIN = long-lasting insecticide-treated net; RDT = rapid diagnostic test. Reproduced from the Pan American Health Organization.^3^

The Pan American Health Organization (PAHO) has outlined its new Diagnosis, Treatment, Investigation and Response (DTI‐R) strategy as a way of “operationalizing in the Americas the concept of malaria surveillance as an intervention, promoted by the WHO in the Global Technical Strategy against Malaria.”^3^ The DTI-R comprises actions triggered by the routine detection of an isolated malaria case or a cluster of cases (Figure 1). The strategy aims to provide access to 1) laboratory diagnosis and 2) prompt malaria treatment, combined with an effort 3) to detect additional cases when an index case has been diagnosed, and 4) to implement vector control measures—mostly indoor residual spraying (IRS) and distribution of long-lasting insecticide-treated bed nets (LLINs)—in the vicinity of passively detected cases (Figure 1,^3^). During the past decade, the Amazonia ICEMR network has addressed each of the four components of the DTI-R strategy and provided evidence that can be translated into real-world interventions.

Our research on laboratory diagnosis of malaria addresses step 1 of the DTI-R strategy. First, we have shown that most parasite carriers in Peru and Brazil harbor low-density parasitemias that are often missed by conventional microscopy.^4^^–^^7^ Mature gametocytes are detected by molecular methods in the vast majority of submicroscopic *P. vivax* carriers,^4^^,^^6^^,^^8^ consistent with their possible role as an infectious reservoir that maintains ongoing malaria transmission.

Next, we and others identified the rapid spread of *Plasmodium falciparum* lineages lacking the histidine-rich protein 2 (HRP2), which severely limits the utility of HRP2-based malaria rapid diagnostic tests (RDTs) in our field sites in the Amazon.^9^^,^^10^ The WHO has developed standardized survey protocols to measure the prevalence of HRP2 deletion, but not all malaria control programs have the capacity to perform the required molecular tests. To fill this gap, ICEMR investigators in Peru joined the international laboratory network set up by the WHO to support the ongoing global mapping of HRP2 deletions (https://www.who.int/malaria/mpac/mpac-mar2017-hrp2-3-deletions-session7.pdf?ua=1).^11^

Malaria infections that are missed by microscopy or RDTs may potentially be detected by field-deployable molecular tests such as simplified protocols for loop-mediated isothermal amplification of nucleic acids.^12^^–^^14^ As examples of policy changes associated with the use of nucleic acid-based diagnosis, we note that a positive molecular test result is now accepted by the Ministry of Health of Brazil as evidence of infection that must trigger antimalarial treatment.^15^ Similarly, molecular tests carried out within the National Network of Public Health Laboratories can confirm malaria diagnosis leading to treatment in Peru.^16^ We note that polymerase chain reaction was recently shown to detect substantially more malaria infections than conventional microscopy in community-wide active case detection rounds in the Loreto region of Peru.^17^ Moreover, the National Plan towards Malaria Elimination in Peru (2021–2030) envisages the gradual implementation of polymerase chain reaction-based and loop-mediated isothermal amplification-based diagnosis in reference laboratories and selected health centers countrywide, consistent with the notion that molecular diagnostics may be incorporated by the national malaria control programs in the Amazon.

Clinical trials are typically outside the scope of the ICEMR research program. However, ICEMR investigators and their partners have contributed to step 2 of the DTI-R strategy by planning, executing, and analyzing clinical studies to monitor the efficacy of antimalarial regimens currently in use in the Amazon. Chloroquine was shown to remain highly efficacious for *P. vivax* malaria in the main transmission hotspot of Brazil,^18^ but mathematical modeling estimates that 11% of *P. vivax* malaria infections will relapse within 12 months despite the routinely prescribed standard low-dose (3.5 mg/kg over 7 or 14 days) treatment with primaquine.^18^ Low cytochrome P450 2D6, or CYP2D6, enzyme activity, which may impair primaquine metabolization and reduce its anti-relapse efficacy,^19^ occurs in 20% to 35% of Amazonians and may account for some primaquine failures.^18^^,^^21^

We have also confirmed the high efficacy of the fixed-dose artesunate–mefloquine combination therapy for *P. falciparum* infection in Brazil,^22^ despite the extensive local use of mefloquine as a monotherapy in the 1990s. This result paved the way for a treatment policy change in Brazil: artesunate–mefloquine was officially reintroduced as a first-line treatment of *P. falciparum* malaria in January 2020.^15^

ICEMR investigators played a leading role in the multicentric DETECTIVE and GATHER trials, which demonstrated that single-dose tafenoquine is as efficacious as the standard low-dose primaquine regimen used across the Amazon to prevent *P. vivax* relapses.^23^^–^^25^ These findings have supported tafenoquine licensing by the U.S. Food and Drug Administration and similar agencies in several malaria-endemic countries, including Peru and Brazil (https://www.keepingthepromisereport.org/case-studies/tafenoquine). Moreover, these studies provided further evidence that suboptimal primaquine doses are routinely prescribed to patients with *P. vivax* malaria who weigh more than 60 kg, leading to more frequent relapses.^23^^–^^25^

Step 3 of the DTI-R strategy comprises reactive case detection and treatment. The ICEMR network generated evidence that supports reactive case detection as a strategy to find additional *P. vivax* infections, most of them asymptomatic, in the vicinity of passively detected index cases in residual malaria settings in the Amazon.^26^ Subclinical *P. vivax* infections missed by routine surveillance tend to be long lasting, and asymptomatic carriers can infect the primary local malaria vector, *Ny. darlingi*, although much less efficiently than symptomatic ones.^27^

Finally, we have identified major challenges for vector control—a key component of the “response” step of the DTI-R strategy. *Nyssorhynchus darlingi* has gradually changed its biting behavior during the past few decades. This vector is now predominantly exophilic and exophagic,^28^^–^^30^ and its biting activity may peak at dusk and dawn or around midnight.^31^ These findings highlight the need for vector control tailored to the changing biting behavior of Amazonian vectors, because outdoor biting and early-evening feeding may undermine the effectiveness of LLINs and IRS in the region.^32^

Larval source management (LSM) is defined as “the targeted management of mosquito breeding sites, with the objective to reduce the number of mosquito larvae and pupae.”^33^ This can be achieved by permanent or recurrent habitat modification, biologic control with natural predators, and chemical or biologic larviciding,^33^ but LSM remains little explored as a supplementary vector control measure in the Amazon. One reason is that vector breeding sites are often not easy to find in densely vegetated areas.^5^ However, we showed that the most productive natural larval habitats may be located accurately using drones equipped with high-resolution multispectral imagery, as distinctive spectral profiles can be characterized for water bodies that are positive for *Ny. darlingi*.^34^

Since the mid-2000s, aquaculture has become an important economic activity in the Amazon, especially in Brazil. We and others have shown that natural and human-made fish-farming ponds are now significant larval habitats across the region.^30^^,^^35^^–^^37^ These easily located breeding habitats are suitable targets for LSM. Accordingly, we have shown that the monthly application of environmentally safe biologic larvicides with extended residual activity, such as commercially available granular formulations of toxins from *Bacillus thuringiensis* serovar *israelensis* and *Lysinibacillus* (formerly *Bacillus*) *sphaericus*,^38^ is an effective way of reducing larval density in fish-farming ponds,^38^^,^^39^ with a potential impact on malaria transmission.^39^ Importantly, these products do not appear to impact biodiversity or interfere with the safety of the water and food sources.^40^^,^^41^

## THE MALARIA ZERO PLAN IN PERU

ICEMR investigators were key contributors to the design and implementation of the malaria elimination program known as Malaria Zero Plan (MZP), which targets the Loreto region, the main transmission area of Peru. Launched in April 2017, the MZP takes a community-level approach to malaria control with the ultimate goal of elimination.^42^ Major components of the Amazonia ICEMR research agenda have been incorporated by the MZP, such as the need to identify and treat asymptomatic parasite carriers and the application of biologic larvicides to supplement core vector control interventions.

**Figure 2.**
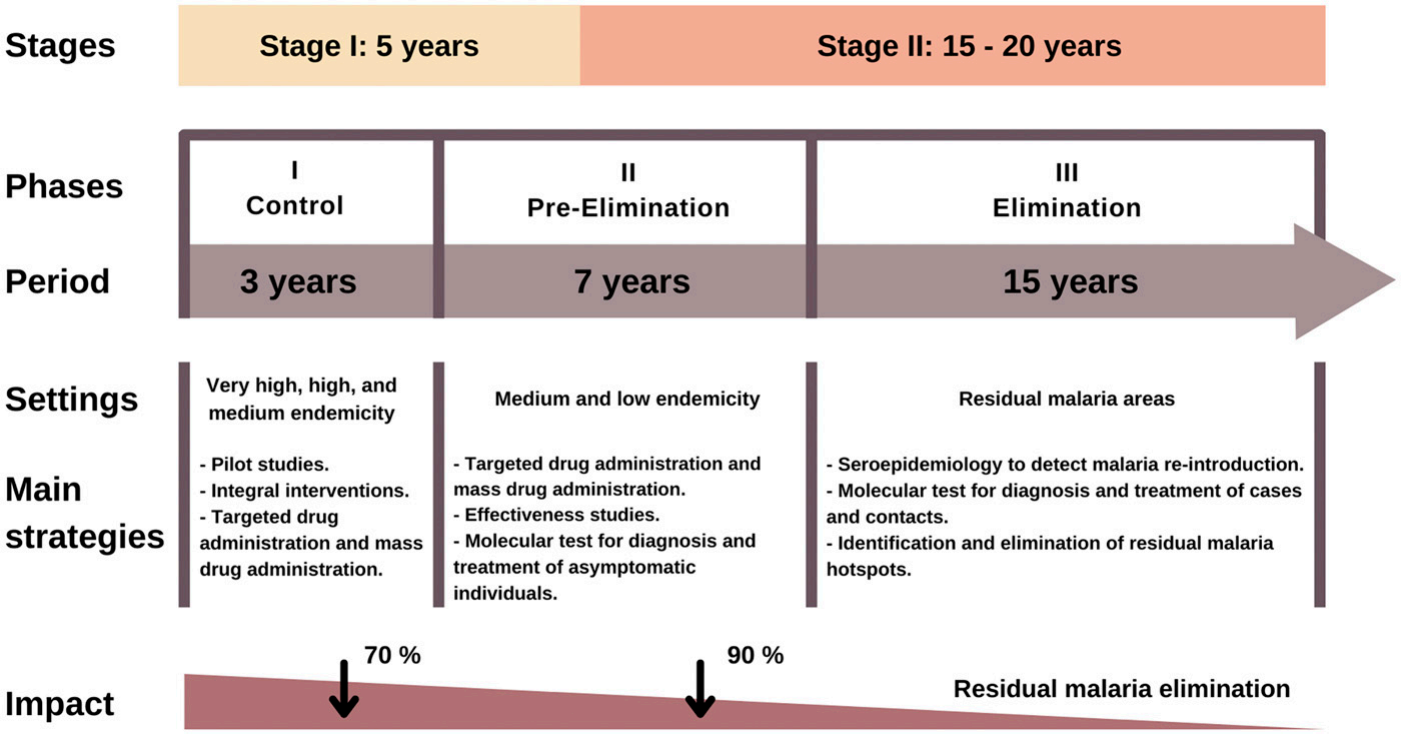
The Malaria Zero Plan strategy of the Ministry of Health of Peru for malaria elimination in the Loreto region. Adapted from Ministry of Health.^39^

The plan comprises three complementary and partially overlapping phases (Figure 2,^42^). The control phase, with an expected duration of 3 years, prioritizes symptomatic infections with the aim of reducing malaria transmission by 70% in settings with very high, high, and moderate endemicity. The next phase aims to eliminate malaria parasites circulating at a regional level by targeting asymptomatic and low-density infections, in addition to symptomatic infections. It will extend over 10 years. The final elimination phase aims to identify and eliminate residual malaria foci and prevent malaria reintroduction. This final phase is expected to last 15 years.

The first phase of MZP targets high-endemicity settings in Loreto. The MZP test-and-treat strategy comprises the provision of RDTs and antimalarials to trained community health promoters to support village-based diagnostics and treatment. These are complemented with vector control measures, such as IRS with the phosphorothioate insecticide pirimiphos–methyl, LLIN distribution, and targeted larviciding. Most community health promoters are members of malaria-endemic Amerindian populations in rural Loreto. Importantly, the Amazonia ICEMR has provided crucial laboratory support to monitor the efficacy of antimalarials and insecticides used in the MZP.^43^

The number of malaria cases recorded in the Loreto region has decreased by 74.5% from 2017 to 2021. The Andoas District in Datem, Marañon Province, was recognized by the PAHO as one of the “Malaria Champions of the Americas” in 2021.^44^ The Malaria Champions of the Americas Award recognizes innovative efforts that contribute significantly toward reducing malaria transmission. Andoas was cited for its steadfast implementation of the MZP, including extensive capacity-building among community health workers, and for maintaining running of the program despite the dramatic effects of the COVID-19 pandemic in the Loreto region.^45^

## INFORMATION AND DATA SHARING

The examples presented here illustrate how the Amazonia ICEMR network has collaborated with policymakers in the Ministries of Health of Peru and Brazil, and PAHO to promote evidence-based malaria control measures across the region. In addition, ICEMR investigators were members of the PAHO Malaria Technical Advisory Group (2015–2021) and the Expert Advisory Committee of the MZP in Peru (2017–2021), and currently serve as technical reviewers of the new National Plan for Malaria Elimination in Peru (2021–2030). The new plan aims to allow continuity of the actions that have been developed in Loreto and extend them to other malaria-endemic regions in Peru.

Communication with study populations exposed to malaria transmission in the Amazon has also been prioritized via regular face-to-face meetings with local stakeholders. Moreover, we have produced a series of podcasts^46^ and short videos^47^^–^^49^ targeted at urban and peri-urban communities where ICEMR activities take place and Internet access is widespread.

The collaboration within the malaria research community, including investigators of several ICEMRs, has been facilitated by extensive sharing of protocols, data, and biologic specimens. Data generated by the Amazonia ICEMR have been made publicly available in open-access online databases such as ClinEpiDB,^50^ PlasmoDB,^51^ and VectorBase,^52^ which are now part of the VEuPathDB resource center^53^ (Table 2). Data sharing has allowed others to reuse data for research and teaching purposes, and to increase the reach and impact of the original studies. Epidemiological data from ClinEpiDB can be used to explore associations between risk factors and malaria, compare diagnostic tests, and so on, whereas serum antibody data shared via PlasmoDB may be used to identify common antigens recognized across geographically disparate areas.^54^ Our extensive data on vector biology in the Amazon can be used to look at abundance of different species and blood meal types and risk of transmission.

**Table 2:** Data generated by the Amazonia International Center of Excellence in Malaria Research made accessible via open-access databases such as ClinEpiDB, PlasmoDB, and VectorBase

Study	Data resource	Data description
Peru cohort	ClinEpiDB Amazonia ICEMR Peru cohort	Human clinical and epidemiological data 2012–15
	PlasmoDB data set DS_4267c95a1c	Human serum antibody levels
	VectorBase MapVEu Project VBP0000527	Mosquito microsatellite data 2012
	VectorBase MapVEu Project VBP0000166	Mosquito blood meal data 2013
Brazil cohort	ClinEpiDB Amazonia ICEMR Brazil cohort	Human clinical and epidemiological data 2010–14
	VectorBase MapVEu Project VBP0000323	Mosquito abundance data 2017

ICEMR = International Center of Excellence in Malaria Research.

## IMPLICATIONS FOR FUTURE RESEARCH

We identified at least four areas where implementation research is urgently needed to translate scientific evidence into malaria control interventions.

First, control and elimination efforts in the Amazon require a quantitative understanding of malaria transmission dynamics for planning, monitoring, and evaluating the effectiveness of interventions. This can be achieved with mathematical models that properly account for risk heterogeneity in communities approaching elimination, where a few high-risk individuals contribute disproportionately to overall malaria burden and onward transmission.^55^ Nevertheless, the potential of mathematical modeling to identify priority targets (e.g., high-risk populations) for interventions remains largely neglected in the Amazon. Building malaria modeling capacity in the region is clearly a top priority.

Second, serological markers can be explored further to identify recent exposure to malaria and support decision making.^56^^,^^57^ For example, the absence of antibody reactivity in children can confirm that malaria transmission has ceased in recent years. Levels of antibodies are useful to map and stratify malaria risk at a regional level, and to assess the effect of control interventions.^58^ Moreover, serology may allow stratification by malaria burden and thus optimize local interventions.^58^ Importantly, whether serological evidence of recent *P. vivax* infection may be used to trigger anti-relapse treatment in public health campaigns remains to be explored. However, surprising little research has addressed the use of novel high-throughput antibody detection methods to guide malaria control policies in the Amazon.

Third, cluster-randomized controlled trials are required to test whether LSM with periodic application of biologic larvicides can supplement core vector control measures to reduce community-wide malaria transmission. Larviciding is well suited to control exophagic and exophilic mosquito vectors in densely populated areas with well-delineated, easy-to-find, and readily accessible breeding sites.^59^ Fish-farming ponds, which are now commonly found in the periphery of cities and towns across the Brazilian Amazon, offer a perfect fit for these criteria.^30^^,^^35^^–^^39^

Last, cluster-randomized controlled trials are needed to evaluate the effectiveness of focal mass drug administration to reduce *P. vivax* transmission in selected transmission hotspots. The pre-elimination phase of the MZP has incorporated targeted antimalarial drug administration as a key strategy in Peru (Figure 2), although locally generated evidence is currently lacking to support its use in public health campaigns across the Amazon.

## CAPACITY-BUILDING AND TRAINING IN RESEARCH CONTEXT

Field and laboratory research carried out by the Amazonia ICEMR investigators has provided graduate and postdoctoral training opportunities for students and scientists from Latin America and other regions across the globe. At the Universidad Peruana Cayetano Heredia in Peru, 10 master of science (MS) candidates, 2 doctoral candidates, and 5 postdoctoral fellows have completed their training since the project onset in 2010; 2 MS candidates, 5 doctoral candidates, and 3 postdoctoral fellows are currently involved in ongoing research projects. At the University of São Paulo in Brazil, 10 MS candidates, 6 doctoral candidates, and 5 postdoctoral fellows have completed their training, and 2 doctoral candidates and 2 postdoctoral fellows are associated with ongoing research. At Fiocruz in Brazil, three MS candidates, two doctoral candidates, and four postdoctoral fellows have participated in the ICEMR projects as part of their training. At SUNY-Albany and the Wadsworth Center, New York State Department of Health, two doctoral candidates and one postdoctoral fellow have completed their training, and one master of public health candidate and one postdoctoral fellow are involved in ongoing research. At the University of California-San Diego and Yale University, a combined total of four MS candidates, four doctoral candidates, and four postdoctoral fellows have carried out onsite training to supplement their education and training in Peru and Brazil. These visiting research experiences were designed to supplement home-country training with exposure to U.S.-based training.

## References

[1] World Health Organization , 2021. World Malaria Report 2021. Geneva, Switzerland: WHO. Available at: https://www.who.int/teams/global-malaria-programme/reports/world-malaria-report-2021. Accessed August 8, 2022.

[2] Ferreira MU Castro MC , 2019. Malaria situation in Latin America and the Caribbean: residual and resurgent transmission and challenges for control and elimination. Methods Mol Biol 2013: 57–70. 31267493 10.1007/978-1-4939-9550-9_4

[3] Pan American Health Organization , 2019. *Stratification of Malaria Based on Risk of Transmission and Elimination of Foci: Region of the Americas*. Available at: https://www3.paho.org/hq/index.php?option=com_docman&view=download&slug=malaria-technical-advisory-group-session-8-2019&Itemid=270&lang=en. Accessed August 8, 2022.

[4] Barbosa S 2014. Epidemiology of disappearing *Plasmodium vivax* malaria: a case study in rural Amazonia. PLoS Negl Trop Dis 8: e3109. 25166263 10.1371/journal.pntd.0003109 PMC4148206

[5] Carrasco-Escobar G Miranda-Alban J Fernandez-Miñope C Brouwer KC Torres K Calderon M Gamboa D Llanos-Cuentas A Vinetz JM , 2017. High prevalence of very-low *Plasmodium falciparum* and *Plasmodium vivax* parasitaemia carriers in the Peruvian Amazon: insights into local and occupational mobility-related transmission. Malar J 16: 415. 29037202 10.1186/s12936-017-2063-x PMC5644076

[6] Rovira-Vallbona E Contreras-Mancilla JJ Ramirez R Guzmán-Guzmán M Carrasco-Escobar G Llanos-Cuentas A Vinetz JM Gamboa D Rosanas-Urgell A , 2017. Predominance of asymptomatic and sub-microscopic infections characterizes the *Plasmodium* gametocyte reservoir in the Peruvian Amazon. PLoS Negl Trop Dis 11: e0005674. 28671944 10.1371/journal.pntd.0005674 PMC5510906

[7] Rosas-Aguirre A 2021. Temporal and microspatial heterogeneity in transmission dynamics of coendemic *Plasmodium vivax* and *Plasmodium falciparum* in two rural cohort populations in the Peruvian Amazon. J Infect Dis 223: 1466–1477. 32822474 10.1093/infdis/jiaa526 PMC8064053

[8] Lima NF Bastos MS Ferreira MU , 2012. *Plasmodium vivax*: reverse transcriptase real-time PCR for gametocyte detection and quantitation in clinical samples. Exp Parasitol 132: 348–354. 22940017 10.1016/j.exppara.2012.08.010 PMC3470767

[9] Gamboa D 2010. A large proportion of *P. falciparum* isolates in the Amazon region of Peru lack *pfhrp2* and *pfhrp3*: implications for malaria rapid diagnostic tests. PLoS One 5: e8091. 20111602 10.1371/journal.pone.0008091 PMC2810332

[10] Rachid Viana GM 2017. Histidine-rich protein 2 (*pfhrp2*) and *pfhrp3* gene deletions in *Plasmodium falciparum* isolates from select sites in Brazil and Bolivia. PLoS One 12: e0171150. 28301474 10.1371/journal.pone.0171150 PMC5354239

[11] World Health Organization, 2017. Protocol for estimating the prevalence of pfhrp2/pfhrp3 gene deletions among symptomatic falciparum patients with false-negative RDT results. Malaria Policy Advisory Committee Meeting. 17–19 October 2017, Geneva, Switzerland Background document for Session 4. Geneva, Switzerland: WHO. Available at: https://www.who.int/docs/default-source/malaria/mpac-documentation/mpac-oct2017-hrp2-deletion-protocol-session4.pdf?sfvrsn=2c9dfaf4_2. Accessed August 8, 2022.

[12] Serra-Casas E 2017. Loop-mediated isothermal DNA amplification for asymptomatic malaria detection in challenging field settings: technical performance and pilot implementation in the Peruvian Amazon. PLoS One 12: e0185742. 28982155 10.1371/journal.pone.0185742 PMC5628891

[13] Nolasco O Infante B Contreras-Mancilla J Incardona S Ding XC Gamboa D Torres K , 2020. Diagnosis of *Plasmodium vivax* by loop-mediated isothermal amplification in febrile patient samples from Loreto, Perú. Am J Trop Med Hyg 103: 1549–1552. 32748776 10.4269/ajtmh.20-0212 PMC7543827

[14] Nolasco O Montoya J Rosales Rosas AL Barrientos S Rosanas-Urgell A Gamboa D , 2021. Multicopy targets for *Plasmodium vivax* and *Plasmodium falciparum* detection by colorimetric LAMP. Malar J 20: 225. 34011373 10.1186/s12936-021-03753-8 PMC8135177

[15] Ministry of Health of Brazil , 2020. *Guidelines for Malaria Therapy in Brazil*. Available at: https://bvsms.saude.gov.br/bvs/publicacoes/guia_tratamento_malaria_brasil.pdf. Accessed August 8, 2021.

[16] Ministry of Health of Peru , 2015. *Norma Técnica de Salud para la Atención de la Malaria y Malaria Grave en el Perú*. Available at: http://bvs.minsa.gob.pe/local/MINSA/4373.pdf. Accessed August 8, 2022.

[17] Moreno-Gutierrez D 2018. Effectiveness of a malaria surveillance strategy based on active case detection during high transmission season in the Peruvian Amazon. Int J Environ Res Public Health 15: 2670. 10.3390/ijerph15122670 PMC6314008 30486449

[18] Ladeia-Andrade S 2019. Monitoring the efficacy of chloroquine–primaquine therapy for uncomplicated *Plasmodium vivax* malaria in the main transmission hot spot of Brazil. Antimicrob Agents Chemother 63: e01965–e18. 30782991 10.1128/AAC.01965-18 PMC6496112

[19] Corder RM de Lima ACP Khoury DS Docken SS Davenport MP Ferreira MU , 2020. Quantifying and preventing *Plasmodium vivax* recurrences in primaquine-untreated pregnant women: an observational and modeling study in Brazil. PLoS Negl Trop Dis 14: e0008526. 32735631 10.1371/journal.pntd.0008526 PMC7423143

[20] Marcsisin SR Reichard G Pybus BS , 2016. Primaquine pharmacology in the context of CYP 2D6 pharmacogenomics: current state of the art. Pharmacol Ther 161: 1–10. 27016470 10.1016/j.pharmthera.2016.03.011

[21] Silvino ACR Kano FS Costa MA Fontes CJF Soares IS de Brito CFA Carvalho LH Sousa TN , 2020. Novel insights into *Plasmodium vivax* therapeutic failure: CYP2D6 activity and time of exposure to malaria modulate the risk of recurrence. Antimicrob Agents Chemother 64: e02056–e19. 32122891 10.1128/AAC.02056-19 PMC7179649

[22] Ladeia-Andrade S de Melo GN de Souza-Lima Rde C Salla LC Bastos MS Rodrigues PT Luz F Ferreira MU , 2016. No clinical or molecular evidence of *Plasmodium falciparum* resistance to artesunate–mefloquine in northwestern Brazil. Am J Trop Med Hyg 95: 148–154. 27068396 10.4269/ajtmh.16-0017 PMC4944679

[23] Llanos-Cuentas A 2014. Tafenoquine plus chloroquine for the treatment and relapse prevention of *Plasmodium vivax* malaria (DETECTIVE): a multicentre, double-blind, randomised, phase 2b dose-selection study. Lancet 383: 1049–1058. 24360369 10.1016/S0140-6736(13)62568-4

[24] Llanos-Cuentas A 2019. Tafenoquine versus primaquine to prevent relapse of *Plasmodium vivax* malaria. N Engl J Med 380: 229–241. 30650326 10.1056/NEJMoa1802537 PMC6657225

[25] Lacerda MVG 2019. Single-dose tafenoquine to prevent relapse of *Plasmodium vivax* malaria. N Engl J Med 380: 215–228. 30650322 10.1056/NEJMoa1710775 PMC6657226

[26] Fontoura PS Finco BF Lima NF de Carvalho JF Jr Vinetz JM Castro MC Ferreira MU , 2016. Reactive case detection for *Plasmodium vivax* malaria elimination in rural Amazonia. PLoS Negl Trop Dis 10: e0005221. 27941968 10.1371/journal.pntd.0005221 PMC5179126

[27] Almeida GG 2021. Asymptomatic *Plasmodium vivax* malaria in the Brazilian Amazon: submicroscopic parasitemic blood infects *Nyssorhynchus darlingi.* PLoS Negl Trop Dis 15: e0009077. 34714821 10.1371/journal.pntd.0009077 PMC8555776

[28] Prussing C Moreno M Saavedra MP Bickersmith SA Gamboa D Alava F Schlichting CD Emerson KJ Vinetz JM Conn JE , 2018. Decreasing proportion of *Anopheles darlingi* biting outdoors between long-lasting insecticidal net distributions in peri-Iquitos, Amazonian Peru. Malar J 17: 86. 29463241 10.1186/s12936-018-2234-4 PMC5819687

[29] Saavedra MP 2019. Higher risk of malaria transmission outdoors than indoors by *Nyssorhynchus darlingi* in riverine communities in the Peruvian Amazon. Parasit Vectors 12: 374. 31358033 10.1186/s13071-019-3619-0 PMC6664538

[30] Rufalco-Moutinho P 2021. Ecology and larval population dynamics of the primary malaria vector *Nyssorhynchus darlingi* in a high transmission setting dominated by fish farming in western Amazonian Brazil. PLoS One 16: e0246215. 33831004 10.1371/journal.pone.0246215 PMC8031405

[31] Oliveira TMP Laporta GZ Bergo ES Chaves LSM Antunes JLF Bickersmith SA Conn JE Massad E Sallum MAM , 2021. Vector role and human biting activity of Anophelinae mosquitoes in different landscapes in the Brazilian Amazon. Parasit Vectors 14: 236. 33957959 10.1186/s13071-021-04725-2 PMC8101188

[32] Moreno M Saavedra MP Bickersmith SA Lainhart W Tong C Alava F Vinetz JM Conn JE , 2015. Implications for changes in *Anopheles darlingi* biting behaviour in three communities in the peri-Iquitos region of Amazonian Peru. Malar J 14: 290. 26223450 10.1186/s12936-015-0804-2 PMC4518648

[33] World Health Organization , 2013. Larval Source Management: A Supplementary Malaria Vector Control Measure: An Operational Manual. Geneva, Switzerland: WHO.

[34] Carrasco-Escobar G 2019. High-accuracy detection of malaria vector larval habitats using drone-based multispectral imagery. PLoS Negl Trop Dis 13: e0007105. 30653491 10.1371/journal.pntd.0007105 PMC6353212

[35] Maheu-Giroux M Casapía M Soto-Calle VE Ford LB Buckeridge DL Coomes OT Gyorkos TW , 2010. Risk of malaria transmission from fish ponds in the Peruvian Amazon. Acta Trop 115: 112–118. 20188688 10.1016/j.actatropica.2010.02.011

[36] dos Reis IC 2015. Contribution of fish farming ponds to the production of immature *Anopheles* spp. in a malaria-endemic Amazonian town. Malar J 14: 452. 26573145 10.1186/s12936-015-0947-1 PMC4647295

[37] dos Reis IC Honorio NA Barros FS Barcellos C Kitron U Camara DC Pereira GR Keppeler EC da Silva-Nunes M Codeco CT , 2015. Epidemic and endemic malaria transmission related to fish farming ponds in the Amazon Frontier. PLoS One 10: e0137521. 26361330 10.1371/journal.pone.0137521 PMC4567347

[38] Fontoura PS da Costa AS Ribeiro FS Ferreira MS Castro MC Ferreira MU , 2020. Field efficacy of VectoMax FG and VectoLex CG biological larvicides for malaria vector control in northwestern Brazil. J Med Entomol 57: 942–946. 31751448 10.1093/jme/tjz220

[39] Fontoura PS Silva MF da Costa AS Ribeiro FS Ferreira MS Ladeia-Andrade S Tonini J Rodrigues PT Castro MC Ferreira MU , 2021. Monthly biological larviciding associated with a tenfold decrease in larval density in fish farming ponds and reduced community-wide malaria incidence in northwestern Brazil. Parasit Vectors 14: 445. 34479606 10.1186/s13071-021-04964-3 PMC8414731

[40] World Health Organization, 1999. Guideline specifications for bacterial larvicides for public health. Report of the WHO Informal Consultation, 28–30 April 1999. Geneva, Switzerland: WHO. Available at: https://www.who.int/publications/i/item/who-cds-cpc-whopes-99.2.

[41] World Health Organization, 1999. Informal consultation on the development of Bacillus sphaericus as a microbial larvicide, Geneva, 7–11 October 1985. Geneva, Switzerland: WHO. Available at: https://apps.who.int/iris/handle/10665/60326.

[42] Ministry of Health of Peru , 2017. *Plan Malaria Cero: Periodo 2017–2021*. Available at: https://docs.bvsalud.org/biblioref/2019/04/965065/rm_244-2017-minsa.pdf. Accessed August 8, 2022.

[43] Quinones ML 2015. Insecticide resistance in areas under investigation by the International Centers of Excellence for Malaria Research: a challenge for malaria control and elimination. Am J Trop Med Hyg 93: 69–78. 26259947 10.4269/ajtmh.14-0844 PMC4574276

[44] Malaria Champions in the Americas 2021 - Andoas, Peru. Video. Available at: https://www.youtube.com/watch?v=yniNikj8APA.

[45] Pan-American Health Organization, 2021. PAHO awards Malaria Champions of the Americas for 2021, launches Multisectoral Action Guide to support fight against the disease. Available at: https://www.paho.org/en/news/5-11-2021-paho-awards-malaria-champions-americas-2021-launches-multisectoral-action-guide. Accessed August 8, 2022.

[46] Radio Moa podcast. 10 episodes. Available at: https://open.spotify.com/show/5Pcglwyj9CCrNWy5tSaO7N. Accessed August 8, 2022.

[47] Diário de Campo: Malária. Video series (6 episodes) [in Portuguese]. Available at: https://www.youtube.com/channel/UCzddVR3V-13u7byxBvkTOlw.

[48] Planeta Ciencia: La investigación que busca eliminar la malaria del Perú. Video [in Spanish]. Available at: https://www.youtube.com/watch?v=lsqASJyl4OQ.

[49] Círculos de investigación combate la Malaria en el Perú. Video [in Spanish]. Available at: https://www.youtube.com/watch?v=gjAgobsWYo0.

[50] Ruhamyankaka E 2019. ClinEpiDB: an open-access clinical epidemiology database resource encouraging online exploration of complex studies. Gates Open Res 3: 1661. 32047873 10.12688/gatesopenres.13087.1 PMC6993508

[51] Aurrecoechea C 2009. PlasmoDB: a functional genomic database for malaria parasites. Nucl Acid Res 37: D539–D543. 10.1093/nar/gkn814 PMC2686598 18957442

[52] Giraldo-Calderón GI 2015. VectorBase: an updated bioinformatics resource for invertebrate vectors and other organisms related with human diseases. Nucl Acid Res 43: D707–D713. 10.1093/nar/gku1117 PMC4383932 25510499

[53] Amos B , 2021. VEuPathDB: the eukaryotic pathogen, vector and host bioinformatics resource center. Nucl Acid Res 50(D1): D898–D911. 10.1093/nar/gkab929 PMC8728164 34718728

[54] Rosado J 2021. Heterogeneity in response to serological exposure markers of recent *Plasmodium vivax* infections in contrasting epidemiological contexts. PLoS Negl Trop Dis 15: e0009165. 33591976 10.1371/journal.pntd.0009165 PMC7909627

[55] Corder RM Ferreira MU Gomes MGM , 2020. Modelling the epidemiology of residual *Plasmodium vivax* malaria in a heterogeneous host population: a case study in the Amazon Basin. PLOS Comput Biol 16: e1007377. 32168349 10.1371/journal.pcbi.1007377 PMC7108741

[56] Rosas-Aguirre A 2020. Anti-MSP-10 IgG indicates recent exposure to *Plasmodium vivax* infection in the Peruvian Amazon. JCI Insight 5: e130769. 10.1172/jci.insight.130769 PMC7030819 31770108

[57] Villasis E 2021. PvMSP8 as a novel *Plasmodium vivax* malaria sero-marker for the Peruvian Amazon. Pathogens 10: 282. 33801386 10.3390/pathogens10030282 PMC7999794

[58] Greenhouse B 2019. Priority use cases for antibody-detecting assays of recent malaria exposure as tools to achieve and sustain malaria elimination. Gates Open Res 3: 131. 31172051 10.12688/gatesopenres.12897.1 PMC6545519

[59] World Health Organization , 2019. Guidelines for Malaria Vector Control. Geneva, Switzerland: WHO. 30844152

